# Trends and Visibility of “Digital Health” as a Keyword in Articles by JMIR Publications in the New Millennium: Bibliographic-Bibliometric Analysis

**DOI:** 10.2196/10477

**Published:** 2019-12-19

**Authors:** Alireza Ahmadvand, David Kavanagh, Michele Clark, Judy Drennan, Lisa Nissen

**Affiliations:** 1 School of Clinical Sciences Faculty of Health Queensland University of Technology Brisbane Australia; 2 School of Psychology and Counselling Faculty of Health Queensland University of Technology Brisbane Australia; 3 School of Advertising, Marketing, and Public Relations QUT Business School Queensland University of Technology Brisbane Australia

**Keywords:** bibliometrics, review literature, JMIR Publications, telemedicine

## Abstract

**Background:**

Digital health has become an advancing phenomenon in the health care systems of modern societies. Over the past two decades, various digital health options, technologies, and innovations have been introduced; many of them are still being investigated and evaluated by researchers all around the globe. However, the actual trends and visibility of peer-reviewed publications using “digital health” as a keyword to reflect the topic, published by major relevant journals, still remain to be quantified.

**Objective:**

This study aimed to conduct a bibliographic-bibliometric analysis on articles published in JMIR Publications journals that used “digital health” as a keyword. We evaluated the trends, topics, and citations of these research publications to identify the important share and contribution of JMIR Publications journals in publishing articles on digital health.

**Methods:**

All JMIR Publications journals were searched to find articles in English, published between January 2000 and August 2019, in which the authors focused on, utilized, or discussed digital health in their study and used “digital health” as a keyword. In addition, a bibliographic-bibliometric analysis was conducted using the freely available Profiles Research Networking Software by the Harvard Clinical and Translational Science Center.

**Results:**

Out of 1797 articles having “digital health” as a keyword, published mostly between 2016 and 2019, 277 articles (32.3%) were published by JMIR Publications journals, mainly in the *Journal of Medical Internet Research*. The most frequently used keyword for the topic was “mHealth.” The average number of times an article had been cited, including self-citations, was above 2.8.

**Conclusions:**

The reflection of “digital health” as a keyword in JMIR Publications journals has increased noticeably over the past few years. To maintain this momentum, more regular bibliographic and bibliometric analyses will be needed. This would encourage authors to consider publishing their articles in relevant, high-visibility journals and help these journals expand their supportive publication policies and become more inclusive of digital health.

## Introduction

### Background

Digital health has become an advancing phenomenon in the health care systems of modern societies [1]. As a keyword, the US Food and Drug Administration defines “digital health” as “a broad scope which includes mobile health (mHealth), health information technology, wearable devices, telehealth and telemedicine, and personalized medicine” [2]. Of note, it is the increasing adoption of “digital health” as a specific keyword, which has shown itself in the utilization of the term by international organizations, such as the World Health Organization [3,4].

Globally, many academics and researchers are increasingly being involved in doing research on, utilizing, evaluating, or taking advantage of the benefits of digital health and its various related technologies for their studies on individuals, populations, or health organizations. This increasing involvement has reflected itself in the utilization of “digital health” as a keyword in published peer-reviewed literature. More specifically, in the past two decades, a growing number and diversity of research projects, study protocols, publications, and dedicated journals have played important roles in the digital health domain [5]. In addition, the empowerment of health care system clients, including patients, and the progressive desire for innovation by industries and enterprises [6] have continued to reinforce the need for valid and trustworthy scientific evidence on digital health for the benefit of public health.

Over the past two decades, various digital health options, technologies, and innovations have been introduced; many of them are still being investigated and evaluated by researchers all around the globe [7]. These research endeavors typically reflect themselves in peer-reviewed publications of various kinds. However, the actual trends and visibility of those publications on digital health, published by major relevant journals, still remain to be quantified in detail.

### Aims

This study aimed to take a more methodical approach to answering this question, by conducting a bibliographic-bibliometric analysis on the publications focused on using “digital health” as a keyword. We evaluated the trends, topics, and citations of research publications in different journals, with the hope to identify, and ultimately help to increase, the share and contribution of major relevant journals in publishing articles on digital health. Thereafter, for the purpose of providing an unbiased comparison among different journals on the trends and visibility of their publications, we conducted detailed subgroup analyses, individualized to specialized journals or journal publishers. This paper summarizes the specific outcomes of our analyses on articles published by JMIR Publications. The main reasons behind focusing on JMIR Publications in this study are the following: (1) JMIR Publications has been an active publisher in the digital health space since 1999, which overlaps entirely with the intended time frame of our study; (2) it has a collection of correlated journals, which covers diverse aspects of digital health research; and (3) it publishes open-access articles, which gives the authors more chances of visibility and knowledge translation and the readers more chances of verifying the results of all analyses.

## Methods

### Rationale Behind Choosing “Digital Health” as a Keyword

On the basis of expert opinions, “digital health” is considered a relatively new term in research publications, as its appearance as a keyword seems to have increased fairly recently in peer-reviewed articles. Before this trend becomes commonplace, keywords such as “Internet research,” “cybermedicine,” “eHealth,” or “mHealth” have been (and are still being) used by authors and editorial boards of various scientific journals, including journals by JMIR Publications.

To address this recency in the adoption of “digital health” as a more common term, we followed a staged, multistep literature search strategy, implemented separately for each journal or journal group or publisher, to ensure that using “digital health” as an identifying keyword does not harm the inclusiveness of numerous options, technologies, and innovations in this space. An effort was made to find the sensitivity of using “digital health” as a keyword in identifying articles that could have otherwise been classified differently under internet search, cybermedicine, mHealth, or similar keywords had “digital health” not been assigned as a keyword by the authors or the databases.

### Literature Search Strategy

The time frame of search was January 2000 to August 2019.

Owing to its open-access nature, we decided to use PubMed database to identify general and specialized journals and find articles published in English language, in which the focus was on using “digital health” as a keyword.

The initial, implicit assumption was that if “digital health” has been mentioned by the authors as a keyword in an article or assigned by the database organizer, for example, as Medical Subject Heading (MeSH)–assigned keyword, the topic of the article will be related to digital health. However, as mentioned above, to reduce the bias in finding relevant articles because of the recency of “digital health” being used as a term, we followed a staged search strategy, which is summarized below.

#### Stage 1

This stage involved finding all articles with “digital health” in their metadata: (1) Searching with only the keyword “digital health” in All Fields to identify all articles in PubMed, which could have the term in their metadata and (2) importing the results to a library in a bibliographic management software.

#### Stage 2

This stage involved identifying keywords/topics/subjects relevant to digital health: (1) Performing a subject bibliography analysis by extracting all author-assigned plus MeSH-assigned keywords, sorted according to their decreasing frequencies of appearance and (2) identifying and refining keywords/topics/subjects relevant to the definition of “digital health,” as provided by Murray et al [8] and later highlighted by Zanaboni et al [7]. The alphabetical list of relevant, refined keywords that we eventually identified appears in Multimedia Appendix 1.

#### Stage 3

This stage involved finding all articles that had used any of the keywords identified in the previous stage: Searching PubMed, using OR between all the keywords from Multimedia Appendix 1.

#### Stage 4

This stage involved finding all articles published by JMIR Publications: Searching with only the keyword “JMIR” in All Fields to identify all articles in PubMed, which were published by JMIR Publications.

#### Stage 5

This stage involved combining stage 3 AND stage 4: Searching PubMed, using OR between all the keywords from Multimedia Appendix 1 AND “JMIR” in All Fields to reidentify all articles published by JMIR Publications, which could have any of the relevant keywords in their metadata.

#### Stage 6

This stage involved comparing the results of stage 5 and stage 4: Determining the difference between the number of articles retrieved in stage 4 and stage 5 to check the inclusiveness of our terms list.

#### Stage 7

This stage involved combining stage 1 and stage 4: (1) After ensuring the sensitivity of our search strategy, on the basis of the outcome of stage 6, we searched with the keyword “digital health” in All Fields AND the keyword “JMIR” in All Fields to identify all articles by JMIR Publications*,* which have the word “digital health” assigned to any of their metadata; (2) importing the results to the same library in the bibliographic management software; and (3) basing the bibliographic-bibliometric analysis on this last group of articles.

A flowchart summarizing the outputs of this staged literature search is available in Multimedia Appendix 2*.*

### Bibliographic–Bibliometric Analysis

For bibliographic management and analysis of the references, we used EndNote X8 (Thompson Reuters Inc) software, mainly its “Subject Bibliography” functionality.

For bibliometric analysis to quantify the trends and visibility of published articles using “digital health” as a keyword, we used one of the free, publicly available Web-based solutions, that is, the Profiles Research Networking (PRN) Software by the Harvard Clinical and Translational Science Center [9]. The details of the methodology behind this specific solution and the range of services the PRN Software provides are explained on its dedicated website. In brief, we used the “Bibliometric Summary Report” functionality of the PRN Web-based software after (1) extracting the PubMed IDs of all articles found in stage 7 of our search strategy and stored in our EndNote library; (2) pasting the IDs onto the PRN Software’s website (in a dedicated box); (3) getting calculations for common metrics, including citation counts and h-index; and (4) analyzing the report metrics and parameters, as per the PRN Software [9] defined in Table 1.

**Table 1:** Bibliometric parameters as provided in the output by the Profiles Research Networking Software in its Bibliometric Summary Report.

Variable	Definition
Num Pubs	Number of recognized PubMed IDs, overall, for each journal, or for each year, as specified in the report subsections
First Year	Earliest article year
Last Year	Latest article year
Avg Authors	Average number of authors per article
Exp Authors	Expected number of authors, matched on journal and year
Ratio Authors	Ratio of the average number of authors to the expected number
Avg Cites All	Average number of times an article has been cited, including self-citations
Avg Cites	Average number of times an article has been cited, not including self-citations
Exp Cites	Expected number of times an article has been cited, not including self-citations, matched on journal and year
Ratio Cites	Ratio of average number of citations (no self-citations) to expected number, matched on journal and year
Exp Cites PT	Expected number of citations (no self-citations), matched on journal, year, and publication type
Ratio Cites PT	Ratio of average number of citations (no self-citations) to expected number, matched on journal, year, and publication type
H-Index	Hirsch-index (using total citations, including self-citations)
M-Index	Hirsch-index divided by the number of years since the first publication
%Pubs	The percentage of the total publications for each journal
Ratio Exp Pubs	The ratio of the number of publications in the field compared with the expected number, matched on year
Num Cites All	For each year, the number of times any article was cited, including self-citations, in that year
Num Cites	For each year, the number of times any article was cited, not including self-citations, in that year
Cum Pubs	For each year, the cumulative number of publications
Cum Cites All	For each year, the cumulative number of times any article was cited, including self-citations
Cum Cites	For each year, the cumulative number of times any article was cited, not including self-citations

## Results

### Overall Findings

Overall, with August 31, 2019 as the last publication date, we found 1797 articles indexed in PubMed, with “digital health” being assigned as one of the keywords in their metadata.

Exporting the keywords from 1797 articles provided a list of 5138 author-assigned and MeSH-assigned keywords, out of which 312 keywords were directly relevant to “digital health” options, technologies, and innovations (Multimedia Appendix 1).

In the same time frame*,* JMIR Publications had 7556 articles indexed in PubMed, mainly in the *Journal of Medical Internet Research* and its sister journals. Using OR between the 312 relevant keywords AND JMIR*,* we were able to identify 7468 (98.8%) of articles by JMIR Publications, an indicator of the high sensitivity of “digital health” as a keyword in an article to represent a diverse range of technologies discussed in their corresponding articles.

Out of the 1797 articles, 277 articles had both characteristics of (1) being published by JMIR Publications and (2) having “digital health” as an assigned keyword. The rest of the bibliographic-bibliometric analysis was performed on these 277 articles.

### Bibliographic Analysis

**Figure 1:**
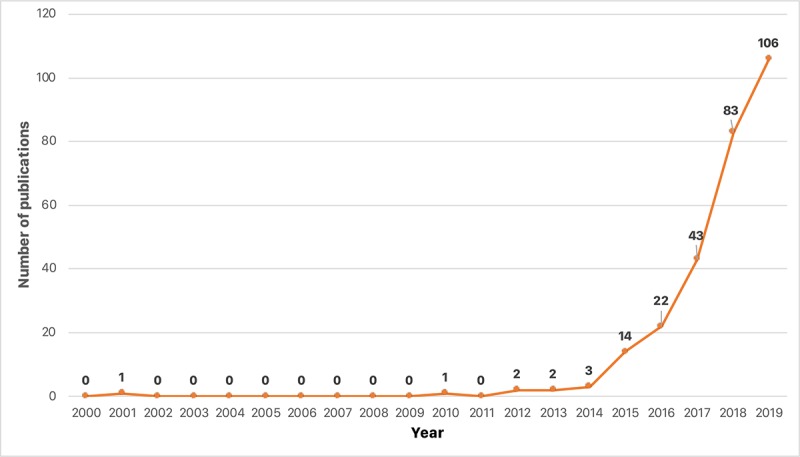
Temporal trend of the number of publications from January 2000 to August 2019 by JMIR Publications, having “digital health” as a keyword.

Figure 1 visualizes the temporal trend of the 277 articles published by JMIR Publications in the study’s time frame. A total of 10 journals by JMIR Publications published most of these articles, the top three being the *Journal of Medical Internet Research* (117/277, 42.2% articles), *JMIR mHealth and uHealth* (57/277, 20.6%), and *JMIR Research Protocols* (41/277, 14.8%).

### Subject Focus of the Articles

Using EndNote’s Subject Bibliography, a total of 1101 MeSH- and author-assigned keywords were extracted for assessing the topics of articles. Table 2 summarizes the top 30 keywords in the published articles and their corresponding number of appearances.

### Bibliometric Analysis

Table 3 summarizes the bibliometric statistics for the published articles having “digital health” as a keyword.

All articles were classified under “medical informatics” as the most frequent field/discipline of focus.

Table 4 summarizes the bibliometric statistics for all articles published between January 2000 and August 2019 by JMIR Publications having “digital health” as a keyword in the study’s time frame (the citation variables have the same meaning as the ones summarized in Table 3).

Table 5 summarizes the yearly cumulative citation statistics for all articles published between January 2000 and August 2019 by JMIR Publications having “digital health” as a keyword (the citation variables have the same meaning as the ones summarized in Tables 2 and 3).

**Table 2:** Cumulative number of appearances for the top 30 keywords, in descending order of appearance, in research articles having “digital health” as a keyword, published from January 2000 to August 2019 in JMIR Publications journals.

Rank	Keyword	Number of appearances
1	mhealth	60
2	Telemedicine	57
3	Internet	42
4	eHealth	37
5	mobile health	36
6	self-management	18
7	mobile phone	15
8	depression	15
9	physical activity	14
10	smartphone	14
11	Mobile Applications	13
12	Chronic Disease	13
13	Social Support	12
14	electronic health records	12
15	psychology	12
16	Health Behavior	11
17	medication adherence	11
18	exercise	10
19	Social Media	10
20	text messaging	9
21	obesity	9
22	education	9
23	mental health	9
24	Health Promotion	8
25	mobile apps	8
26	diabetes	8
27	Diabetes Mellitus	8
28	telehealth	7
29	Cell Phone	7
30	Health Personnel	7

**Table 3:** Bibliometric summary statistics for all articles published between January 2000 and August 2019 by JMIR Publications having “digital health” as a keyword.

Variable	Value
Num Pubs^a^	277
First Year^b^	2001
Last Year^c^	2019
Avg Authors^d^	6.007
Exp Authors^e^	6.212
Ratio Authors^f^	0.967
Avg Cites All^g^	2.848
Avg Cites^h^	2.354
Exp Cites^i^	1.451
Ratio Cites^j^	1.623
Exp Cites PT^k^	1.688
Ratio Cites PT^l^	1.394
H–Index^m^	12
M–Index^n^	1.091

^a^Num Pubs: number of recognized PubMed IDs, overall, for each journal, or for each year, as specified in the report subsections.

^b^First Year: earliest article year.

^c^Last Year: latest article year.

^d^Avg Authors: average number of authors per article.

^e^Exp Authors: expected number of authors, matched on journal and year.

^f^Ratio Authors: ratio of the average number of authors to the expected number.

^g^Avg Cites All: average number of times an article has been cited, including self-citations.

^h^Avg Cites: average number of times an article has been cited, not including self-citations.

^i^Exp Cites: expected number of times an article has been cited, not including self-citations, matched on journal and year.

^j^Ratio Cites: Ratio of average number of citations (no self-citations) to expected number, matched on journal and year.

^k^Exp Cites PT: Expected number of citations (no self-citations), matched on journal, year, and publication type.

^l^Ratio Cites PT: Ratio of average number of citations (no self-citations) to expected number, matched on journal, year, and publication type.

^m^H-Index: Hirsch-index (using total citations, including self-citations).

^n^M–Index: Hirsch-index divided by the number of years since the first publication.

**Table 4:** Bibliometric statistics for all articles published between January 2000 and August 2019 by JMIR Publications having “digital health” as a keyword.

Journal	Num Pubs^a^ (%Pubs)^b^, n (%)	First Year^c^	Last Year^d^	Avg Cites^e^	Exp Cites^f^	Ratio Cites^g^	Exp Cites PT^h^	Ratio Cites PT^i^
*Journal of Medical Internet Research*	117 (42.2)	2001	2019	3.79	2.02	1.88	2.34	1.62
*JMIR mHealth and uHealth*	57 (20.6)	2014	2019	1.11	1.24	0.90	1.23	0.90
*JMIR Research Protocols*	41 (14.8)	2014	2019	1.12	0.65	1.74	0.65	1.72
*JMIR Mental Health*	12 (4.3)	2016	2019	1.83	1.69	1.09	1.49	1.24
*JMIR Medical Informatics*	10 (3.6)	2015	2019	0.50	0.62	0.81	0.59	0.86
*JMIR Diabetes*	8 (2.9)	2017	2019	0.00	0.04	0.00	0.03	0.00
*JMIR Public Health and Surveillance*	7 (2.5)	2016	2019	0.29	0.77	0.37	0.79	0.36
*JMIR Formative Research*	7 (2.5)	2017	2019	0.00	0.00	1.00	0.00	1.00
*JMIR Human Factors*	5 (1.8)	2017	2019	0.40	0.43	0.94	0.43	0.93
*JMIR Serious Games*	4 (1.4)	2013	2018	16.75	7.87	2.13	15.66	1.07

^a^Num Pubs: Number of recognized PubMed IDs, overall, for each journal, or for each year, as specified in the report subsections.

^b^%Pubs: The percentage of the total publications for each journal**.**

^c^First Year: Earliest article year.

^d^Last Year: Latest article year.

^e^Avg Cites: Average number of times an article has been cited, not including self-citations.

^f^Exp Cites: Expected number of times an article has been cited, not including self-citations, matched on journal and year.

^g^Ratio Cites: Ratio of average number of citations (no self-citations) to expected number, matched on journal and year.

^h^Exp Cites PT: Expected number of citations (no self-citations), matched on journal, year, and publication type.

^i^Ratio Cites PT: Ratio of average number of citations (no self-citations) to expected number, matched on journal, year, and publication type.

**Table 5:** Cumulative citation statistics for all articles published between January 2000 and August 2019 by JMIR Publications having “digital health” as a keyword, by year.

PubYear^a^	Num Pubs^b^	Num Cites All^c^	Num Cites^d^	Cum Pubs^e^	Cum Cites All^f^	Cum Cites^g^
2018	83	309	253	171	789	652
2017	43	249	197	88	480	399
2016	22	108	94	45	231	202
2015	14	55	42	23	123	108
2014	3	32	31	9	68	66
2013	2	19	19	6	36	35
2012	2	8	7	4	17	16
2011	0	6	6	2	9	9
2010	1	1	1	2	3	3
2009	0	1	1	1	2	2
2008	0	0	0	0	0	0
2007	0	0	0	0	0	0
2006	0	0	0	0	0	0
2005	0	0	0	0	0	0
2004	0	1	1	1	1	1
2003	0	0	0	0	0	0
2001	1	0	0	1	0	0
2000	0	0	0	0	0	0

^a^Authors excluded 2019 from this table as the cumulative citations might be incomplete because of the study time frame being up to August 2019.

^b^Num Pubs: Number of recognized PubMed IDs, overall, for each journal, or for each year, as specified in the report subsections.

^c^Num Cites All: For each year, the number of times any article was cited, including self-citations, in that year.

^d^Num Cites: For each year, the number of times any article was cited, not including self-citations, in that year.

^e^Cum Pubs: For each year, the cumulative number of publications.

^f^Cum Cites All: For each year, the cumulative number of times any article was cited, including self-citations.

^g^Cum Cites: For each year, the cumulative number of times any article was cited, not including self-citations.

## Discussion

### Principal Findings

Both trends and visibility of research publications containing “digital health” in their keywords and published by JMIR Publications journals increased dramatically, especially over the past 2 to 3 years, with more than two-third of the articles being published in 2018 and 2019. This important finding shows how “digital health” is becoming a mainstream theme and an established terminology in peer-reviewed publications.

The *Journal of Medical Internet Research* had the highest number of articles and longest duration of publication in this time frame, among all the journals of JMIR Publications. This reflects the overall aim and willingness of the editorial board to lead in peer review, and ultimately in the publication, of the manuscripts that are focused on digital health to disseminate their ideas and research results. It may also reflect improvement in the methodologies of the published articles [10], which might have made them strong and robust enough to be accepted for publication in the JMIR Publications journals.

Interestingly, “mHealth” and “mobile health” as specific keywords, appeared in 96 out of 277 articles (34.6%), followed by “Telemedicine” and “Internet,” both appearing in 57 (20.5%) and 42 (15.2%) articles, respectively. In addition, there appeared to be cumulatively repetitive or redundant keywords, either author-assigned or MeSH keywords (eg, “mobile phone,” “smartphone,” “Cell Phone,” “Mobile Applications,” and “mobile apps”), all appearing with different frequencies in collective articles. We decided to present these keywords as raw as possible in Table 2 to show how different some of these keywords still are, in appearing in the topics of research manuscripts. This highlights the fact that the authors and/or manually indexing databases, such as National Library of Medicine (NLM), can take advantage of the conceptual trends and assign more appropriate keywords to improve their accuracies in retrieving and combining relevant search results.

The dramatic increase in the cumulative number of citations over the study years is a helpful indicator of the overall interest in referring to the articles pertaining to the keyword “digital health.” Moreover, an H-index of 12, plus an average number of citations of all articles being >1.6 times more than the expected number of citations, highlights the increasing interest in referring to articles on digital health.

Expectedly, “medical informatics” was found to be the most frequent field/discipline of focus in research publications having “digital health” as a keyword. This finding, in addition to considering the *Journal of Medical Internet Research* as ranking first in the category “Medical Informatics” in *Journal Citations Report*, highlights the suitability of this study to be presented to the audiences of the journal.

The *Journal of Medical Internet Research* and *JMIR Research Protocols* had the highest ratio of average number of citations (no self-citations) to expected number, matched on journal, year, and publication type (PT). This highlights the higher visibility of research publications in the abovementioned journals.

### Increasing the Accuracy of Interpreting Bibliometric Outputs

We followed the hints provided by the PRN Software team [9] to increase the reliability and validity of interpreting the bibliometric outputs:

1. The PRN Software compares the average number of authors per article and the average number of times the articles have been cited with an expected value, which is “the averages of all articles in PubMed, matched on journal and year of publication” [9]. To control for the various PTs (eg, Journal Article, Review, and Editorial), the software also calculates “PT” expected values. As PubMed may assign multiple PTs to the same article, articles are matched on all PTs for calculating the PT expected values.
2. In addition, if self-citations are included in the analysis subsections, they are explicitly being noted.
3. To determine the field/discipline of a specific journal, the NLM assigns Broad Journal Heading values to the journal, which are MeSH terms, summarizing the overall subjects of that journal. Similar to PT, a journal can be assigned to multiple Broad Journal Headings; consequently, a single publication of that journal might be listed more than once in the output tables about filed/discipline, causing the Num Pubs field to add up to more than the total number of publications. This was not the case in our analysis as all the journals by JMIR Publications were classified under Medical Informatics by the NLM.

### Limitations

Our study focused on English language–based journals that were indexed in PubMed as a freely available database and published by JMIR Publications. PubMed is not essentially a citation-tracking database. However, solutions such as the bibliometric solution that we used in our methodology, that is, the PRN Software by the Harvard Clinical and Translational Science Center, have been developed, which provide bibliometric outputs on PubMed-indexed articles. Other citation-based databases, specifically subscription-based bibliometric databases, such as Scopus and Web of Science, could be included in future research projects to expand the scope of this analysis.

Another main reason behind focusing only on PubMed, apart from being freely available to the public, was that PRN Software only accepts PubMed IDs for citation analysis. This held us back from using other bibliographic databases as they could not have any PubMed ID for non-PubMed-indexed journals.

In addition, the citation metrics by PRN Software were coming from one publicly available free data source and were limited to commonly used parameters. For the provision of a comprehensive bibliometric outlook on publications by JMIR Publications having the keyword “digital health,” other citation databases and metrics could also be utilized in future studies.

### Conclusions

The reflection of “digital health” in JMIR Publications journals has been on the rise over the past few years. More comprehensive and comparative bibliographic and bibliometric analyses, with broader ranges of keywords to include eHealth, mHealth, and similar concepts, would be needed to visualize whether “digital health” continues to remain a rising keyword in the future or not.

## Appendix

Multimedia Appendix 1 List of refined keywords relevant to digital health, to reduce bias in the search strategy.

Multimedia Appendix 2 Search flowchart.

## Abbreviations

HDR: higher degree research
MeSH: Medical Subject Headings
NLM: National Library of Medicine
PRN: Profiles Research Networking
PT: publication type
QUT: Queensland University of Technology
