# Supplementary material for: Trends and Visibility of “Digital Health” as a Keyword in Articles by JMIR Publications in the New Millennium: Bibliographic-Bibliometric Analysis
Source: J Med Internet Res. 2019 Dec 19;21(12):e10477. doi: 10.2196/10477 (PMC6940860; doi:10.2196/10477)
Supplement: Multimedia Appendix 1 [file jmir_v21i12e10477_app1.docx]

Trends and Visibility of ‘Digital Health’ as a Keyword in Research Publications in the New Millennium: A Bibliographic–Bibliometric Analysis on Articles by *JMIR Publications*

**Appendix 1 – List of refined keywords relevant to digital health, to increase the specificity of the search strategy**

Accelerometer

Access to Information

activity monitor

activity tracker

activity trackers

Algorithms

app

apps

artificial intelligence

Attitude to Computers

augmented reality

Automation

Big data

bioinformatics

Biomedical informatics

Biomedical Technology

Biosensing Techniques

Blockchain

blogging

Cell Phone

Chatbots

clinical decision support

Cloud Computing

Computer Communication Networks

computer literacy

Computer Security

computer simulation

computer systems

Computer technology

Computer-Assisted Instruction

computers

computers, handheld

computing methodologies

Connectivity

consumer health informatics

Consumer Health Information

consumer wearable technology

cybersecurity

data

Data Accuracy

Data analysis

data governance

Data linkage

data mining

Data privacy

data quality

data science

Data sharing

data visualization

Databases, Factual

Decision Support Techniques

Deep learning

digital

digital biomarkers

digital diabetes

digital divide

digital health

digital health care

digital health intervention

digital health interventions

Digital health kits

digital health literacy

digital health literacy skills

digital health monitoring

digital health services

digital health social network

digital health technologies

digital health technology

Digital Identity

Digital image management

Digital Imaging and Communications in Medicine

digital information

digital intervention

digital interventions

digital media

digital medicine

digital medicine system

digital medicines

digital opinion leader

digital orthopedics

digital pathways

digital patient

Digital pen & paper

digital pills

Digital psychosis

digital research

Digital resources

digital revolution

Digital sociology

digital strategy

digital technologies

Digital technology

Digital therapeutics

digital therapy

digital transformation

digital weight loss intervention

Digitization

Dpp4bit

E-health

E-healthcare

E-learning

e-patient

e-TB Manager

e-therapy

eConsult

eCPAT

Education, Distance

eHealth

eHealth behavior

eHealth implementation

eHealth Literacy

eHealth review

eLearning

Electronic Data Processing

electronic health

electronic health

electronic health record

electronic health records

electronic health records

Electronic immunisation registry

Electronic Mail

electronic medical records

Electronic Nicotine Delivery Systems

electronic patient records

electronic patient-controlled health record

electronic personal health record

Electronic Prescribing

electronic prescription claims

electronic survey

Electronic tablet

Electronics

Electronics, Medical

email

Enterprise Image Repository

Enterprise imaging platform

Enterprise PACS

eSource

Facebook

Facebook app

Fitbit

Fitbit Zip

fitness app

fitness tracker

fitness trackers

game-based learning

games and gamification

Games, Experimental

Games, Recreational

gamification

gaming technology

Genomics

Geographic Information Systems

gerontechnology

Google

Hashtags

health APIs

Health apps

Health Care Technology

health data

Health data cooperatives

health games

health informatics

Health information

Health Information Exchange

health information management

Health information sources

Health information system

Health Information Systems

Health information technologies

Health information technologies

health information technology

health IT

Health IT safety

health monitoring

Health Records, Personal

Health technology

Healthcare technology

Home monitoring

Hospital Information Systems

Image Interpretation, Computer-Assisted

Image Processing, Computer-Assisted

infodemiology

informatics

Information and Communications Technology

Information Dissemination

information management

information quality

Information Seeking Behavior

information services

information storage and retrieval

Information systems

Information Technology

Insulin Infusion Systems

internet

Internet Access

Internet of Things

Interoperability

m-health

machine learning

Medical Informatics

Medical Informatics Applications

Medical Records Systems, Computerized

medical technology

medtech

mHealth

Mobile

mobile app

mobile application

Mobile Applications

mobile apps

Mobile devices

Mobile health

Mobile health

mobile phone

Mobile phones

My Health Record

Neural Networks

Nursing informatics

Online

Online communities

Online information

online support groups

Online Systems

open data

patient access to records

Patient Discharge

Patient portal

patient portals

patient web portals

PCEHR

pedometer

personal health information

personal health record

personal health records

personalised health

Personalized medicine

persuasive technology

Photography

Point-of-Care Systems

precision medicine

Professional-Patient Relations

protocol

Ptsd

public health informatics

quantified-self

Radiology Information Systems

Reminder Systems

remote consultation

remote monitoring

Remote Sensing Technology

Self-Help Devices

self-tracking

Semantics

sensor

sensors

Signal Processing, Computer-Assisted

smartglasses

Smartphone

Smartphone app

smartphone applications

smartphones

smartwatch

social marketing

social media

social network

Social Networking

social networking sites

Social networks

Software

Software Design

Startups

Step counts

superusers

Systems Integration

tablet computers

Tablets

technology

technology acceptance

Telecare

Telecommunications

Telehealth

Telemedicine

Telemetry

Telephone

Telepsychiatry

Telerehabilitation

television

text message

text messages

text messaging

Therapy, Computer-Assisted

Twitter

User experience

user-centered design

User-Computer Interface

Video Games

Video Recording

videoconferencing

virtual care

virtual reality

wearable

wearable device

wearable devices

Wearable Electronic Devices

wearable sensor

Wearable sensors

Wearable technology

Wearables

web 2.0

Web medicine

wireless

Wireless Technology
