# Supplementary material for: Trends and Visibility of “Digital Health” as a Keyword in Articles by JMIR Publications in the New Millennium: Bibliographic-Bibliometric Analysis
Source: J Med Internet Res. 2019 Dec 19;21(12):e10477. doi: 10.2196/10477 (PMC6940860; doi:10.2196/10477)
Supplement: Multimedia Appendix 2 [file jmir_v21i12e10477_app2.docx]

Trends and Visibility of ‘Digital Health’ as a Keyword in Research Publications in the New Millennium: A Bibliographic–Bibliometric Analysis on Articles by *JMIR Publications*

**Appendix 2 – Search flowchart**


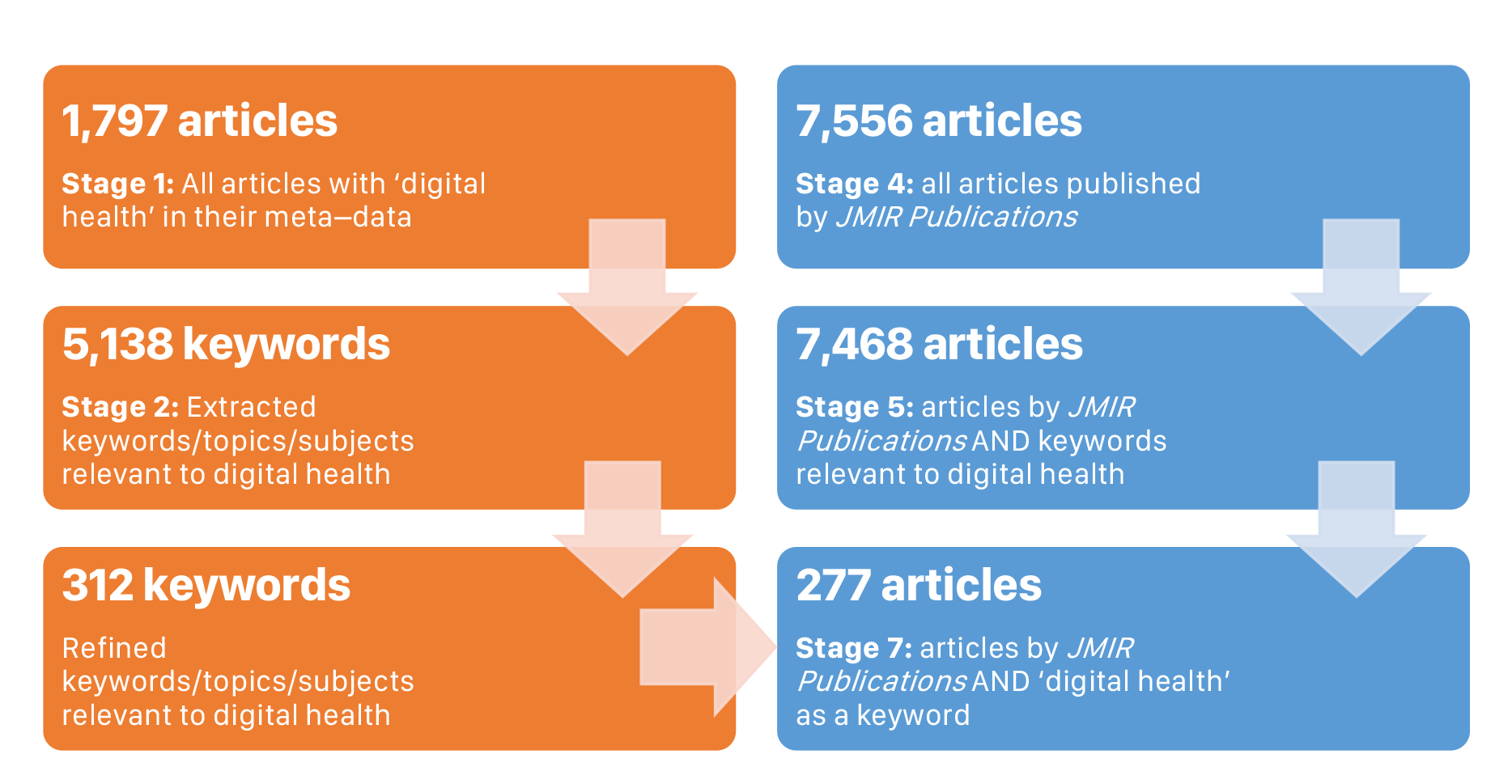


* Note: Stages 3 and 6 not shown for simplicity. Please refer to the main text for elaboration.
